# Identifying risk factors associated with postpartum depression: a retrospective case-control study

**DOI:** 10.1186/s12884-026-09932-2

**Published:** 2026-09-17

**Authors:** Ricarda Volz, Volker Arnd Coenen, Juan Carlos Baldermann, Máté Dániel Döbrössy

**Affiliations:** 1 Laboratory of Stereotaxy and Interventional Neurosciences, Department of Stereotactic and Functional Neurosurgery, University Freiburg Medical Center, Freiburg im Breisgau, Germany; 2 Faculty of Medicine, University of Freiburg, Freiburg, Germany; 3 Department of Stereotactic and Functional Neurosurgery, University Freiburg Medical Center, Freiburg im Breisgau, 79106 Germany; 4 Faculty of Biology, University of Freiburg, Freiburg im Breisgau, 79104 Germany; 5 Department of Psychiatry and Psychotherapy, University Freiburg Medical Center, Freiburg im Breisgau, 79104 Germany

**Keywords:** Depression, Postpartum, Mental health, Risk factors, Social support, Prenatal stress

## Abstract

**Background:**

Postpartum depression (PPD) is a common complication following childbirth, affecting approximately 10–20% of women. Despite its prevalence, PPD remains underdiagnosed and insufficiently addressed in routine perinatal care, particularly in large cohorts from German-speaking countries. The study aimed to provide a comprehensive analysis of biological and psychosocial risk factors across the perinatal period that contribute to the onset of PPD.

**Methods:**

A retrospective case-control study was conducted via an online survey with nearly 1,000 recent mothers from Germany, Austria, and Switzerland who had given birth within the last five years. Participants were classified as Cases or Controls based on whether they had experienced PPD and were asked about a broad range of biological and psychosocial factors. Correlational analyses were used to identify factors associated with PPD.

**Results:**

A total of 975 women were included in the final analysis. Based on self-reported PPD and Edinburgh Postnatal Depression Scale scores, participants were classified into the Case (*n* = 470) and Control group (*n* = 505). Factors with moderate significance generally or during pregnancy, included a history of depression, perceived support during pregnancy, financial and environmental stress during pregnancy, birth satisfaction and traumatic birth experiences. Factors showing moderate significant results postpartum (within the first six weeks after birth) included sleep disturbances, support by the partner, and medical or financial stress. Strongly significant factors present during pregnancy comprised prenatal depression, perceived lack of support, loneliness, general stress and happiness, while strong significant risk factors postpartum included medical support, support by family and friends, and general as well as environmental stress.

**Conclusions:**

The findings highlight significant associations between psychosocial factors and PPD, particularly stress, lack of perceived social support, loneliness during pregnancy, and negative or traumatic birth experiences. Importantly, clinically relevant depressive symptoms were already present during pregnancy in a substantial proportion of women, suggesting that depressive symptoms may already occur before childbirth rather than being restricted to the postpartum period. These results highlight the importance of early screening and preventive strategies during pregnancy, particularly within routine antenatal care. Large-scale anonymous online surveys may further contribute to improved detection of unrecognised PPD cases and help address underdiagnosis of the disorder.

**Supplementary Information:**

The online version contains supplementary material available at https://doi.org/10.1186/s12884-026-09932-2.

## Background

Postpartum depression (PPD) is a form of clinical depression that affects approximately 10–20% of individuals following childbirth [1]. It is characterised by persistent depressive symptoms such as sadness, anhedonia, fatigue, and anxiety, which may substantially impair functioning and the ability to care for oneself or the infant [1, 2]. PPD is highly underdiagnosed, with around 50% of cases being unrecognised [3], and can have substantial consequences for mothers, infants, partners, and families [1]. These include impaired maternal-infant bonding, reduced breastfeeding rates, developmental and behavioural problems in the child, increased maternal substance use, and elevated risk for suicidal ideation [4–6]. Typically, PPD occurs within the first weeks postpartum, but symptoms can manifest anytime within the first year, while approximately 11.5% develop symptoms already during pregnancy [4, 7, 8]. Recurrence is common, particularly in women with early-onset PPD [4].

PPD develops through a complex interplay of biological and psychosocial factors across the perinatal period [2]. Biological factors thought to contribute to the onset of PPD include hormonal changes, neurotransmitter imbalances, and inflammation [3]. Psychological risk factors include a history of depression and chronic stress, while social factors, such as lack of support and cultural expectations, also play a critical role [3]. However, the detailed pathogenesis of PPD is not well understood and possible risk factors contributing to the development of PPD require further investigation [1, 2]. Given the potentially severe consequences of untreated PPD for the mother and the child, early identification and intervention are essential to improve symptoms and reduce the risk of progression to more severe psychiatric outcomes [2].

Various diagnostic tools are available to identify individuals with PPD, including the Edinburgh Postnatal Depression Scale (EPDS) and other validated screening tools such as the Center for Epidemiologic Studies of Depression instrument (CES-D) or the Postpartum Depression Screening Scale (PDSS) [9, 10]. Among these, the EPDS is the most commonly used tool, with a high sensitivity and specificity in detecting PPD [10, 11].

Despite the growing literature on PPD, most previous studies have focused on single risk factors, such as hormonal changes, psychosocial stress, or social support, without analysing the influence of biological compared to psychosocial factors [12, 13]. Thus, the relative importance of different predictors remains unclear [14]. To address this gap, the present study analysed a wide range of risk factors associated with PPD to gain a comprehensive understanding of their relative impact. Additionally, large-scale studies from German-speaking populations are limited [15, 16]. Therefore, this study aimed to identify key biological and psychosocial risk factors for PPD in recent mothers from Germany, Austria, and Switzerland using a retrospective online case-control survey, which is well suited for exploring associations between a broad range of potential risk factors and PPD in heterogeneous populations. The survey analysed biological and psychosocial risk factors that occurred before, during, or after pregnancy to examine whether specific factors are associated with PPD during pregnancy or in the postpartum period.

## Methods

### Study design and participants

The study used a retrospective online case-control design, which is suitable for studying multiple potential risk factors associated with the onset of PPD [17]. Since the objective was to collect data on a wide range of potential predictors, an online survey format was selected, allowing the collection of large datasets [18, 19].

The target population included women from Germany, Austria, and Switzerland who had given birth at least once in the last five years. Participants were recruited via parenting apps, social media groups, and newsletters from mother support organisations, forming a convenience sample [20, 21]. They further represented a purposive non-probability sample, since inclusion criteria ensured that only individuals meeting the study’s purpose were eligible [22]. This included participants who had given birth within the last five years, possessed adequate knowledge of German, and had access to an internet-enabled device. Exclusion criteria were survey completion in less than five or more than twenty minutes, or unwillingness to provide informed consent [22]. Furthermore, Participants giving birth within the last six weeks were excluded because PPD may not yet have manifested during this period. Consequently, women who would later develop PPD could have been incorrectly classified as controls, resulting in misclassification bias.

### Survey development

The study followed the principles of CHERRIES (The Checklist for Reporting Results of Internet E-Surveys) [22], as well as recommendations by Ball [21] and Regmi et al. [23], to ensure scientific standards for online surveys. The survey was structured into sub-sections. Participants first received a detailed introduction, including the purpose of the study and an explanation of PPD. The estimated completion time was noted as approximately ten minutes. A warning about potential trigger topics was provided due to the sensitive nature of the survey. Then, participants were informed about voluntary participation and the right to withdraw at any time and were offered a chance to win a gift voucher as an incentive for completing the survey [22]. Subsequently, participants were classified into Case and Control groups using adaptive questioning [1]. Those reporting a previous diagnosis of PPD were assigned to the Case group. Participants without a known history of PPD completed an adapted retrospective version of the EPDS [11] to screen for potential previously unrecognised cases. As participants retrospectively reported symptoms occurring after childbirth within the previous five years, item wording was slightly modified to reflect retrospective symptom reporting rather than symptoms experienced during the past seven days, as assessed in the original EPDS. Response options were standardised across items to ensure consistency, while all ten original EPDS items were retained. No items were added or removed. A cutoff score of ≥ 13, consistent with prior literature [10], was applied to classify probable cases. The adapted version of the EPDS, embedded in the study survey, is provided in Additional File 2.

The main survey assessed demographic information and a wide range of biological and psychosocial risk factors occurring before and during pregnancy, as well as in the first six weeks postpartum [3, 12, 24]. Participants who indicated that they had not experienced PPD answered in regard to their most recent pregnancy, while participants with a history of PPD reported on their most recent PPD episode. The questions assessing biological and psychosocial risk factors were developed by analysing recent literature on risk factors linked to PPD. This literature-informed approach aimed to ensure comprehensive coverage of relevant predictors. An overview of the included risk factors and corresponding references is provided in the Additional file 1.

The survey contained single- and multiple-choice questions (nominal scale) as well as five-point Likert scale questions (ordinal scale). Each question included an alternative option (e.g., “I don’t know”) to allow abstention without generating missing data. No forced-choice method was used, and participants were able to continue the survey even if they didn’t answer all questions [21, 22]. The survey was performed as an open survey, meaning that anyone who met the inclusion criteria and accessed the survey link could participate [22].

For validation, the survey was pretested to assess clarity, relevance, and comprehensiveness, and minor adjustments were made accordingly [21]. Additionally, technical functionality was tested [22]. The final version was provided in German and English, with the English version being a direct translation of the German survey [23]. It contained 35 pages, with 35–37 questions depending on PPD status. The complete version of the survey is available in the Additional file 2.

### Data collection and ethics

Data were collected via the secure web-based platform SoSciSurvey (SoSci Survey GmbH, Munich, Germany), between May 17th and July 5th, 2024.

The survey was fully anonymous: no personal identifiers, IP addresses, or cookies were collected [22]. No interventions were performed, and therefore no trial registration was required. Participation was voluntary, and electronic informed consent was obtained before survey access, in accordance with the International Committee of Medical Journal Editors (ICMJE) recommendations [25]. The study was conducted using fully anonymised data. The Ethics Committee of the University of Freiburg reviewed the study and confirmed that no formal ethical approval was required, as all analysed data were fully anonymised.

### Data analysis

Data were exported from SoSciSurvey to Microsoft Excel. Only participants that completed at least 70% of the survey and met all eligibility criteria were included. Then, completion and response rates as well as average completion time were calculated [22]. Descriptive analyses were performed for demographic variables.

Participants were grouped based on their responses to the question “Did you experience PPD in the last five years?”. Those who answered “yes” were classified as individuals with PPD. Participants who answered “no” or “I don’t know” completed an adapted version of the EPDS [11]. Those scoring ≥ 13 were considered to represent probable cases with previously unrecognised postpartum depressive symptoms and were assigned to the group “EPDS ≥ 13”, whereas participants scoring < 13 formed the group “EPDS < 13” [10]. For the main analysis, participants reporting a history of PPD were combined with those in the “EPDS ≥ 13” group to form a single Case group, representing women with either self-reported PPD or probable postpartum depressive symptomatology. The “EPDS < 13” group served as the Control group. This approach was chosen to account for potentially undiagnosed cases of PPD and to reduce the risk of misclassification associated with relying exclusively on self-reported diagnoses. Given that PPD is frequently under-recognised [3], only adding women reporting a previous diagnosis to the case group could have resulted in women with clinically relevant depressive symptoms being incorrectly classified as controls. Participants with EPDS scores ≥ 13 were therefore included in the Case group to depict a broader spectrum of postpartum depressive symptomatology in this population-based sample. In addition, combining both groups ensured an adequate sample size, which had been calculated a priori assuming a strong expected effect size (degrees of freedom = 2; Cramer’s V = 0.35) [26], thereby providing sufficient statistical power to detect meaningful associations and enabling a clear comparison between individuals with and without PPD [27]. To assess the robustness of this classification, sensitivity analyses were performed using alternative case definitions. Specifically, women with self-reported PPD and women with EPDS scores ≥ 13 were analysed separately against the control group to determine if the pattern of associations differed depending on case definition.

After grouping, responses regarding the analysed risk factors were examined. Single-choice and multiple-choice items were presented as percentages, while Likert-scale items were summarized as mean scores per group. Graphs were generated to illustrate differences between the Case and Control groups, focusing on risk factors that showed significant differences.

As the survey primarily generated nominal and ordinal data, measures of variability such as standard deviation were not applicable [26].

### Statistical analysis

To examine if certain risk factors were significantly associated with the onset of PPD, chi-square tests of independence were performed [26, 28]. Thereby, Chi-square tests were intentionally chosen because they could be applied consistently across nominal, multiple-choice, and Likert-scale variables, allowing direct comparison of results across all analysed factors. A significance level of α = 0.05 was applied. To account for multiple testing, Bonferroni correction was applied based on the number of examined risk factors in the primary analyses. As 53 variables, including statistically significant and non-significant factors, were analysed, the significance threshold was adjusted to α = 0.05/53 = 0.000943. The same corrected significance threshold was subsequently used for the sensitivity analyses. As these analyses assessed the same predefined risk factors using alternative case definitions rather than testing additional independent hypotheses, they were interpreted as robustness analyses and not included in a separate multiple-testing correction framework.

For single- or multiple-choice questions, the chi-square test was performed by using absolute frequencies. For Likert scale questions, responses were grouped by frequency counts (1–5) and added into contingency tables. After performing all chi-square tests for this study, the results were interpreted based on the p-value [28].

Additionally, Cramer’s V was calculated to examine the strength of significant results (weak, moderate or strong). Effect sizes were interpreted according to established thresholds adjusted for the degrees of freedom of the contingency table [29] (see Additional file 3).

All statistical analyses were performed using Python in Jupyter Notebook [30–32]. Custom scripts were developed to calculate chi-square statistic, critical value, p-value, degrees of freedom and Cramer’s V effect size for each analysis [29]. The full code used for the analysis is provided in an OSF respiratory [33]. All analyses were performed on a univariate level. No multivariable adjustment was applied, as the aim of the study was exploratory identification of potential risk factors.

### Data availability

The dataset and analysis code supporting the conclusions of this article are available in the OSF repository [33] under embargo and will be made publicly accessible upon publication. During the embargo period, the data are available from the corresponding author upon reasonable request.

## Results

### General data

The survey was available for 49 days, with a total of 1,722 clicks registered on the survey link. Of these, 1,209 individuals started the survey, and 1,042 completed at least 70% of it. This led to a completion rate of 86.1% and a response rate of 60.5%.

After applying exclusion criteria, 20 datasets were excluded for completion times under five minutes, 7 for over twenty minutes, and 37 participants were removed for having given birth less than six weeks prior. Three additional datasets were excluded due to repetitive “I don’t know” responses, resulting in a final sample size of 975 participants. The average completion time was 9 min 54 s.

### Demographic data

The final sample (*n* = 975) consisted predominantly of women living in Germany (96%), aged between 30 and 34 years (42%), and with a high level of education (53% holding a university degree). Most participants were employed (86%). Detailed demographic information is provided in the Additional file 4.

### Determining case and control groups

**Fig. 1:**
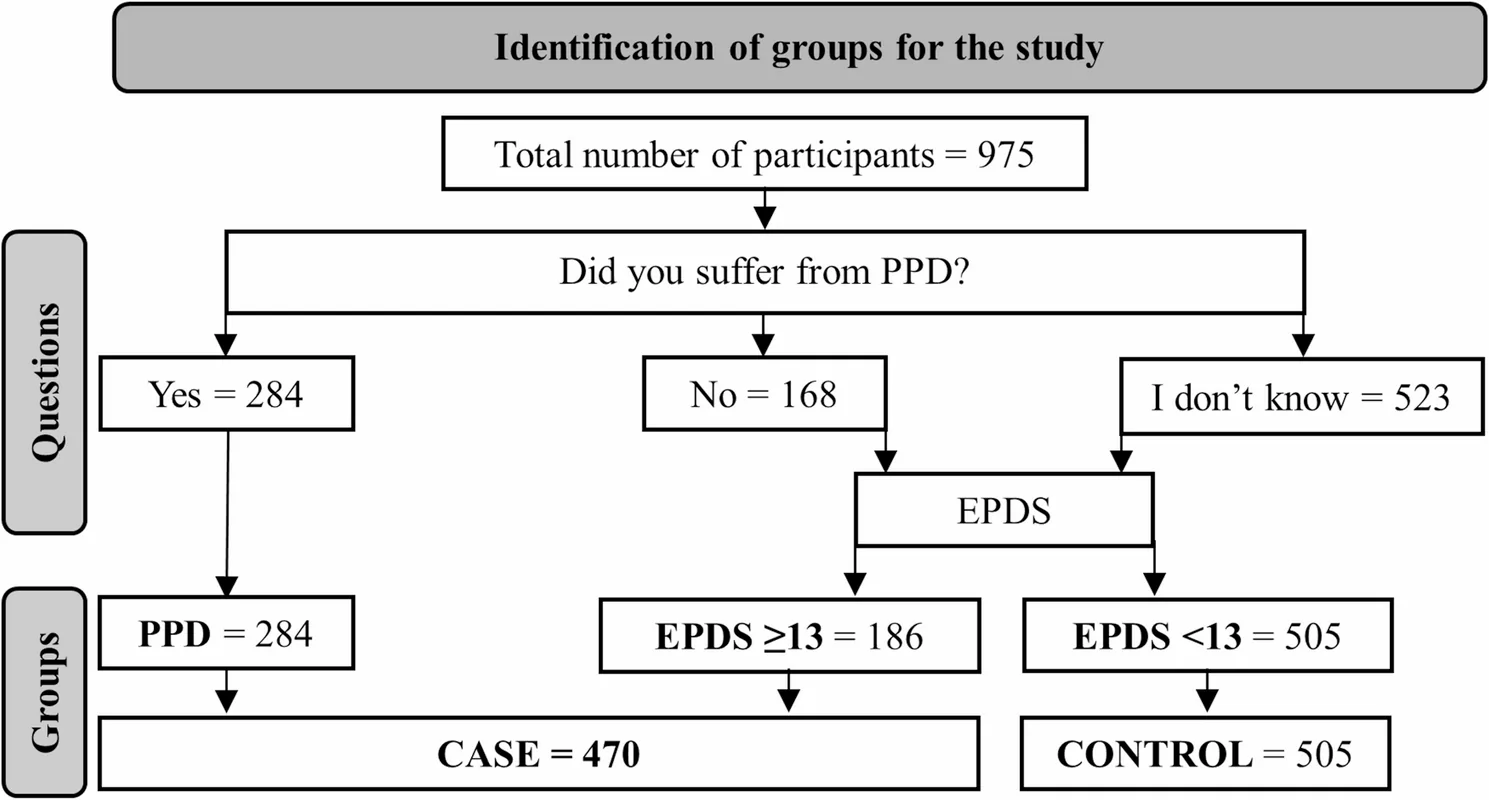
Flow diagram of participant classification. Flow diagram illustrating the classification of participants (*n* = 975) into the Case and Control groups based on self-reported PPD and EPDS scores. The diagram also shows the subdivision of the Case group into women with self-reported PPD and women with EPDS scores ≥ 13 used for the sensitivity analyses

Of all participants, 284 reported having experienced PPD within the last five years, 168 were unsure, and 523 reported no PPD history. Based on this self-report and EPDS scores, participants were subsequently assigned to the Case and Control groups. The final sample included 470 participants in the Case group and 505 in the Control group. Based on the final sample sizes of the Case and Control groups, a post-hoc power analysis, using *G*Power 3.1 [34], confirmed that the achieved sample provided > 99% power (α = 0.05, two-tailed). An overview of separating participants into Case and Control is illustrated in a flow diagram (Fig. 1).

### Risk factor analysis

To identify risk factors of PPD, all survey questions were examined. Statistically significant findings are presented in detail below. Additional associations showing no or weak statistical significance are briefly reported for completeness. All reported associations remained robust after Bonferroni correction.

### History of depression

**Fig. 2:**
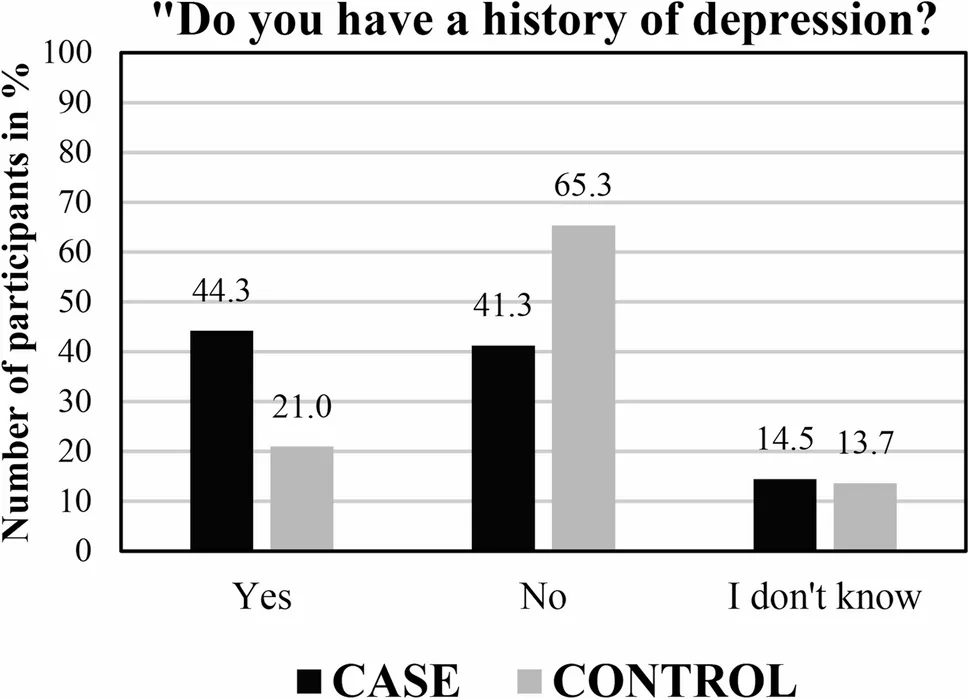
History of depression of the participants. An illustration of the proportion of participants in the Case and Control groups reporting a previous history of depression. Responses are presented as percentages within each group. A higher proportion of participants with PPD (Case group) reported a previous depressive episode compared to the Control group

As shown in Fig. 2, a larger proportion of participants in the Case group reported a previous history of depression (44.3%) compared to the Control group (21.0%). The proportion of participants uncertain about a possible history of depression was similar across both groups (Case = 14.5%; Control = 13.7%). Chi-square analysis (Table 1) indicated a moderate significant association between a history of depression and the Case group (*p* < 0.05; Cramer’s V = 0.26).

**Table 1:** Association between history of depression and PPD

Variable:	χ²	*p*-value	df	Cramer’s V
Chi-square test: history of depression	67.27	2.47E-15	2	0.26

Association between a history of depression and PPD in the Case and Control groups were assessed using chi-square tests.

*χ*² chi-square statistic, *df* degrees of freedom, effect sizes are reported as Cramer’s V

### Prenatal depression

**Fig. 3:**
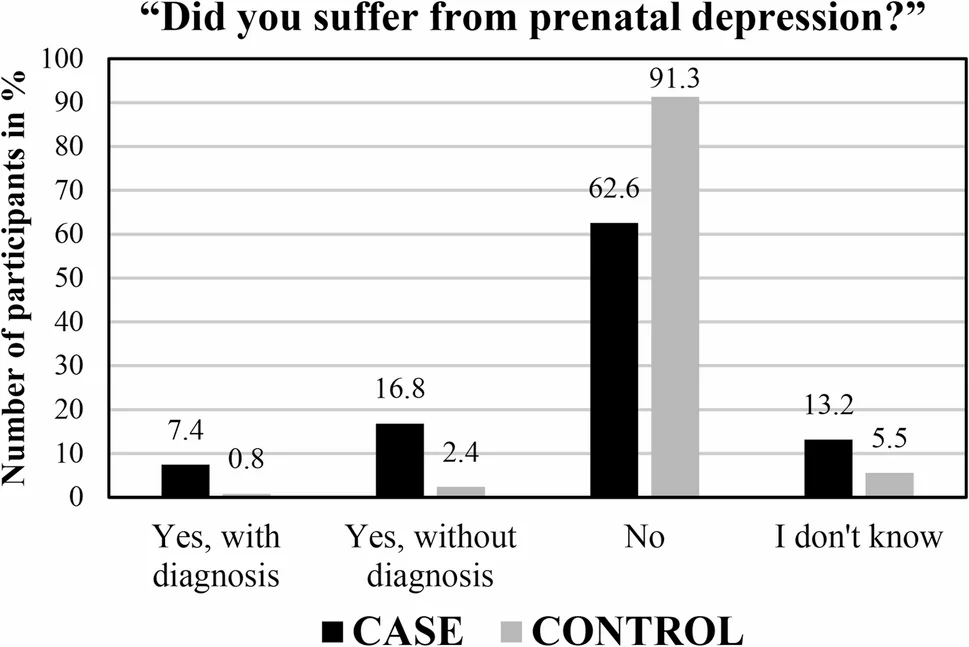
Prenatal depression. The proportion of participants in the Case and Control groups reporting a history of prenatal depression is illustrated. Responses are shown as percentages within each group. A higher proportion of participants with PPD reported officially as well as self-diagnosed prenatal depressive symptoms compared to the Control group

As shown in Figs. 3 and 7.4% of all participants in the Case group reported a clinical diagnosis of prenatal depression, compared to 0.8% in the Control group. Self-reported prenatal depression was also higher in the Case group (16.8%) than in the Control group (2.4%). A smaller proportion of the Case group indicated no history of prenatal depression (62.6% vs. 91.3%), and 13.2% of the Case group were unsure, compared to 5.5% of Controls. Chi-square analyses (Table 2) indicated strong significant differences between the Case and Control group for prenatal depression (*p* < 0.05; Cramer’s V = 0.35).

**Table 2:** Association between prenatal depression and PPD

Variable:	χ²	*p*-value	df	Cramer’s V
Chi-square test: prenatal depression	122.66	2.07E-26	3	0.35

Association between prenatal depression and PPD in the Case and Control groups were assessed using chi-square tests

*χ*² chi-square statistic, *df* degrees of freedom, effect sizes are reported as Cramer’s V

### Support during pregnancy

**Fig. 4:**
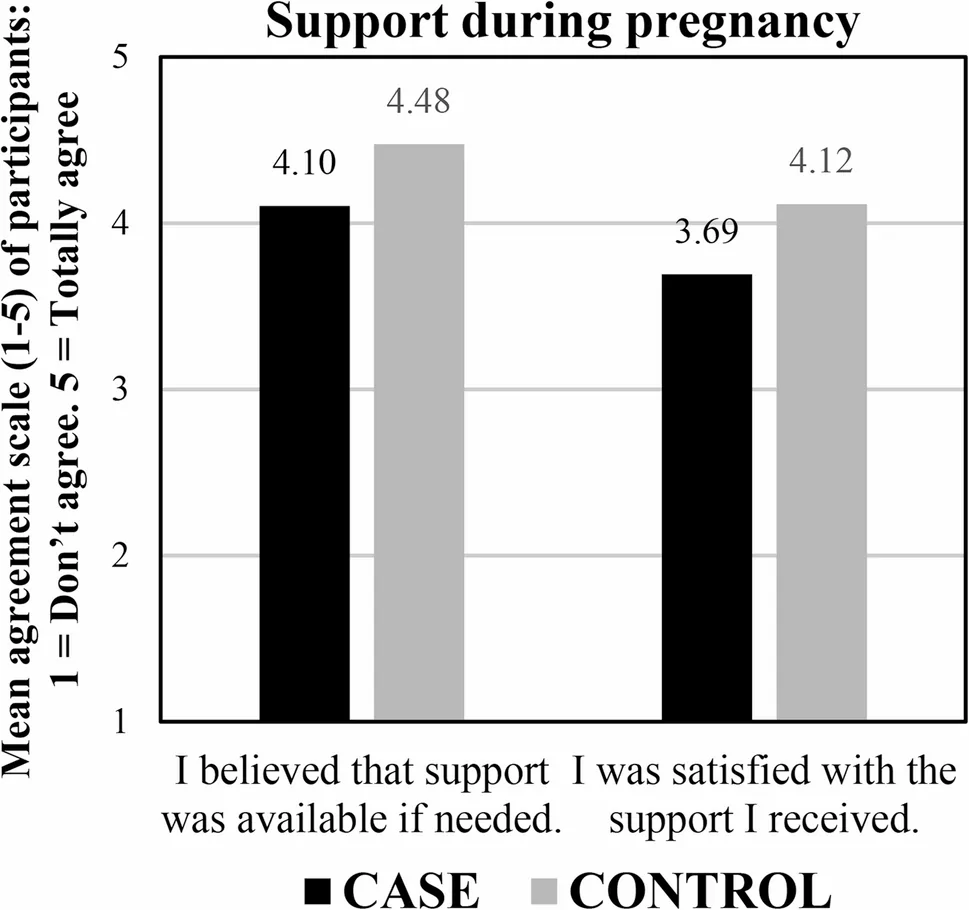
Mean agreement scores regarding support during pregnancy. The mean agreement scores for statements assessing perceived and received support during pregnancy among the Case and Control groups is shown from 1–5 (1 = don’t agree, 5 = totally agree). Higher scores indicate stronger agreement with each statement

Support during pregnancy was assessed using Likert-scale questions. The statements showing significant differences between the Case and Control groups included: “I believed that support was available if needed” (perceived support) and “I was satisfied with the support I received” (received support). For both statements, participants in the Control group reported higher agreement scores (Fig. 4).

The chi-square analysis revealed a strong significant association for perceived availability of support (*p* < 0.05; Cramer’s V = 0.23) and moderate significant association for satisfaction with received support (*p <* 0.05; Cramer’s V = 0.18). Table 3 summarises the corresponding chi-square results.

**Table 3:** Association between support during pregnancy and PPD

Variable	χ²	*p*-value	df	Cramer’s V
Chi-square test: “I believed that support was available if needed”	36.32	2.5E-07	4	0.19
Chi-square test: “I was satisfied with the support I received”	33.74	8.42E-07	4	0.19

Associations between perceived and received support during pregnancy and PPD in the Case and Control groups were assessed using chi-square tests. Analyses include the two statements: “I believed that support was available if needed” and “I was satisfied with the support I received”

*χ*² chi-square statistic, *df* degrees of freedom, effect sizes are reported as Cramer’s V

### Chronic stress during pregnancy

**Fig. 5:**
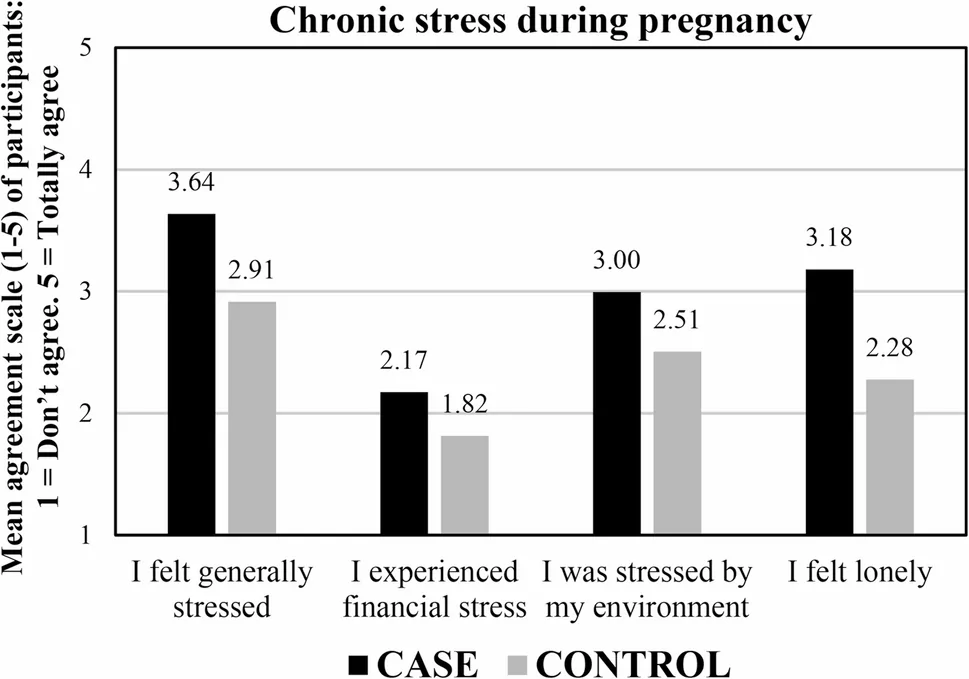
Mean agreement scores regarding chronic stress during pregnancy. The mean agreement scores for statements assessing chronic stress during pregnancy among the Case and Control groups are shown on a scale from 1 to 5 (1 = don’t agree, 5 = totally agree). Assessed statements included “I felt generally stressed,” “I experienced financial stress,” “I was stressed by my environment,” and “I felt lonely.” Higher scores indicate stronger agreement with each statement

Sudden and chronic stress during pregnancy were assessed. Only chronic stress, rated on a Likert scale ranging from 1 (don’t agree) to 5 (totally agree), showed significant results. As shown in Fig. 5, participants in the Case group reported higher average agreement scores across all statements compared to the Control group, including “I felt generally stressed”, “I experienced financial stress”, “I was stressed by my environment” and “I felt lonely”. Chi-square analyses (Table 4) confirmed moderate to strong significant differences among the Case and Control groups in regard to chronic stress during pregnancy. The statements “I felt generally stressed” (*p* < 0.05, Cramer’s V = 0.27), “I was stressed by my environment” (*p* < 0.05, Cramer’s V = 0.18), and “I felt lonely” (*p* < 0.05, V = 0.31), all showed strong significant associations. Financial stress showed only moderate significance (*p* < 0.05, *V* = 0.16).

**Table 4:** Association between chronic stress during pregnancy and PPD

Variable	χ²	*p*-value	df	Cramer’s V
Chi-square test: “I felt generally stressed”	70.62	1.68E-14	4	0.27
Chi-square test: “I experienced financial stress”	25.1	4.84E-05	4	0.16
Chi-square test: “I was stressed by my environment”	32.75	1.34E-06	4	0.18
Chi-square test: “I felt lonely”	96.44	5.62E-20	4	0.32

Associations between stress during pregnancy and PPD in the Case and Control groups were assessed using chi-square tests. Analysed statements include: “I felt generally stressed”, “I experienced financial stress”, “I was stressed by my environment” and “I felt lonely”

χ² chi-square statistic, *df*  degrees of freedom, effect sizes are reported as Cramer’s V

### Happiness during pregnancy

**Fig. 6:**
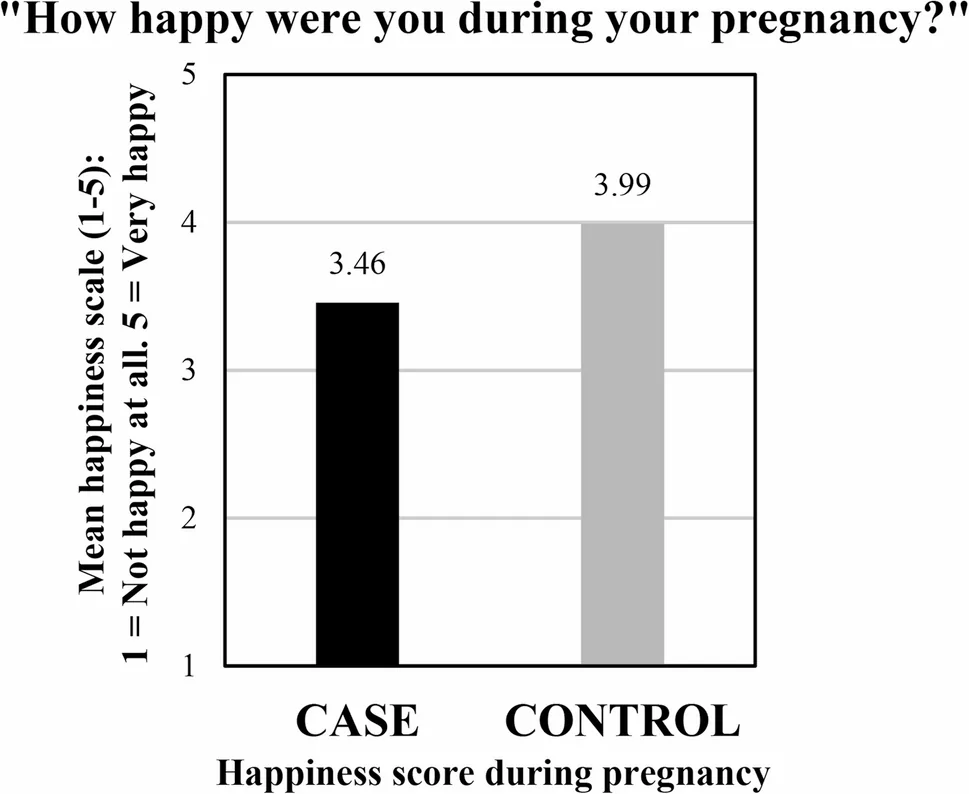
Perceived happiness during pregnancy. The average scores for happiness during pregnancy comparing Case and Control, ranging from 1 (not happy at all) to 5 (very happy). Higher scores indicate a higher level of self-reported happiness during pregnancy

A Likert scale question assessed how happy participants felt during pregnancy, ranging from 1 (not happy at all) to 5 (very happy). Participants in the Case group reported lower happiness scores (mean = 3.46) compared to the Control group (mean = 3.99) (Fig. 6). The chi-square analysis (Table 5) indicated a significant difference between the Case and Control groups in regard to happiness during pregnancy (*p* < 0.05), with a strong effect size (Cramer’s V = 0.32).

**Table 5:** Association between happiness during pregnancy and PPD

Variable	χ²	*p*-value	df	Cramer’s V
Chi-square test: happiness during pregnancy	67.05	9.52E-14	4	0.26

Association between happiness during pregnancy and PPD in the Case and Control groups were assessed using chi-square tests

χ² chi-square statistic, *df* degrees of freedom, effect sizes are reported as Cramer’s V

### Birth satisfaction

**Fig. 7:**
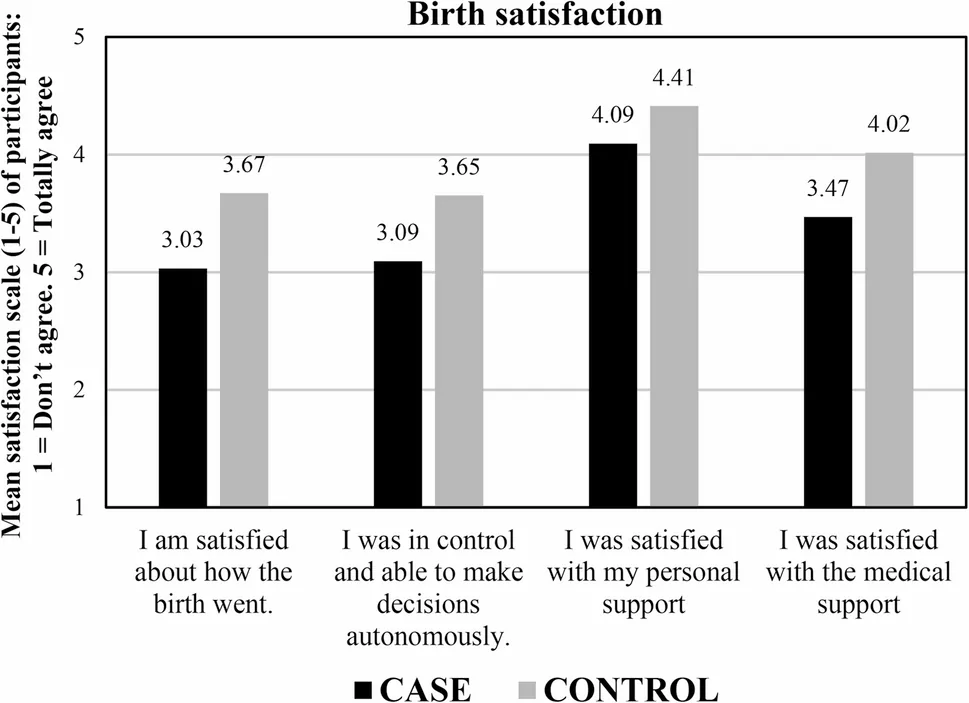
Mean satisfaction scores regarding birth satisfaction. The average agreement scores to statements about birth satisfaction for Case and Control groups. Statements include: “I am satisfied about how the birth went”, “I was in control and able to make decisions autonomously”, “I was satisfied with my personal support”, and “I was satisfied with the medical support”. Responses ranging from 1 (don’t agree) to 5 (totally agree), with higher scores indicating stronger agreement with each statement

Satisfaction with childbirth was assessed using Likert scale questions, rating from 1 (don’t agree) to 5 (totally agree). Overall, participants in the Case group reported lower birth satisfaction across all statements compared to the Control group (including “I am satisfied with how the birth went”, “I was in control and able to make decisions autonomously”, “I was satisfied with my personal support”, and “I was satisfied with the medical support” (Fig. 7).

As shown in Table 6, the chi-square test indicated a moderate significant association for all statements including “I am satisfied with how the birth went” (*p* < 0.05, Cramer’s V = 0.23), “I was in control and able to make decisions autonomously” (*p* < 0.05, Cramer’s V = 0.23), “I was satisfied with my personal support” (*p* < 0.05, Cramer’s V = 0.16), and “I was satisfied with the medical support” (*p* < 0.05, Cramer’s V = 0.22).

**Table 6:** Association between birth satisfaction and PPD

Variable	χ²	*p*-value	df	Cramer’s V
Chi-square test: “I am satisfied about how the birth went”	52.29	1.2E-10	4	0.23
Chi-square test: “I was in control and able to make decisions autonomously”	56.39	1.66E-11	4	0.24
Chi-square test: “I was satisfied with my personal support”	27.32	1.72E-05	4	0.17
Chi-square test: “I was satisfied with the medical support”	47.11	1.45E-09	4	0.22

Associations between birth satisfaction and PPD in the Case and Control groups were assessed using chi-square tests. Analysed statements include: “I am satisfied about how the birth went”, “I was in control and able to make decisions autonomously”, “I was satisfied with my personal support”, and “I was satisfied with the medical support”

*χ*² chi-square statistic, *df* degrees of freedom, effect sizes are reported as Cramer’s V

### Traumatic birth

**Fig. 8:**
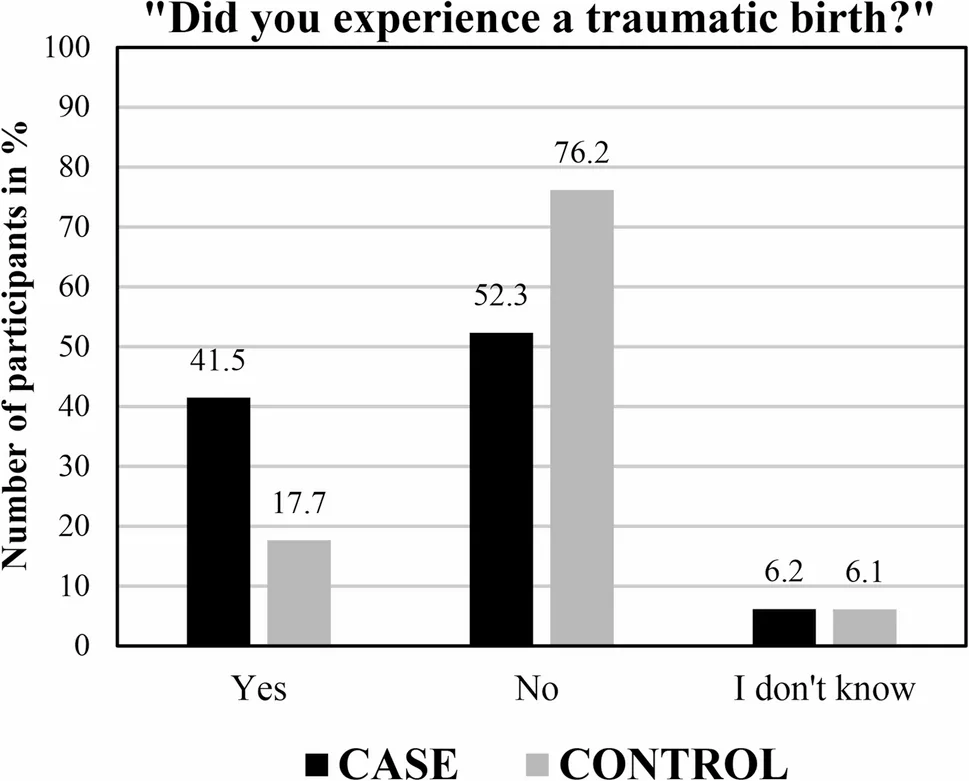
Traumatic birth. The proportion of participants in the Case and Control groups reporting a traumatic birth is presented. Responses are shown as percentages within each group

Participants were asked whether they perceived their birth experience as traumatic. Overall, 41.5% of individuals in the Case group reported having experienced a traumatic birth, compared to only 17.7% in the Control group. In contrast, 52.3% of cases and 76.2% of Controls indicated that they did not perceive their birth as traumatic (Fig. 8).

The chi-square test confirmed a significant difference between perceived birth trauma among the Case and Control groups, with a moderate effect size (*p* < 0.05, *V* = 0.25) (Table 7).

**Table 7:** Association between a traumatic birth and PPD

Variable	χ²	*p*-value	df	Cramer’s V
Chi-square test: traumatic birth	68.76	1.17E-15	2	0.27

Association between experiencing a traumatic birth and PPD in the Case and Control groups were assessed using chi-square tests

*χ*² chi-square statistic, *df* degrees of freedom, effect sizes are reported as Cramer’s V

### Sleep disturbances postpartum

**Fig. 9:**
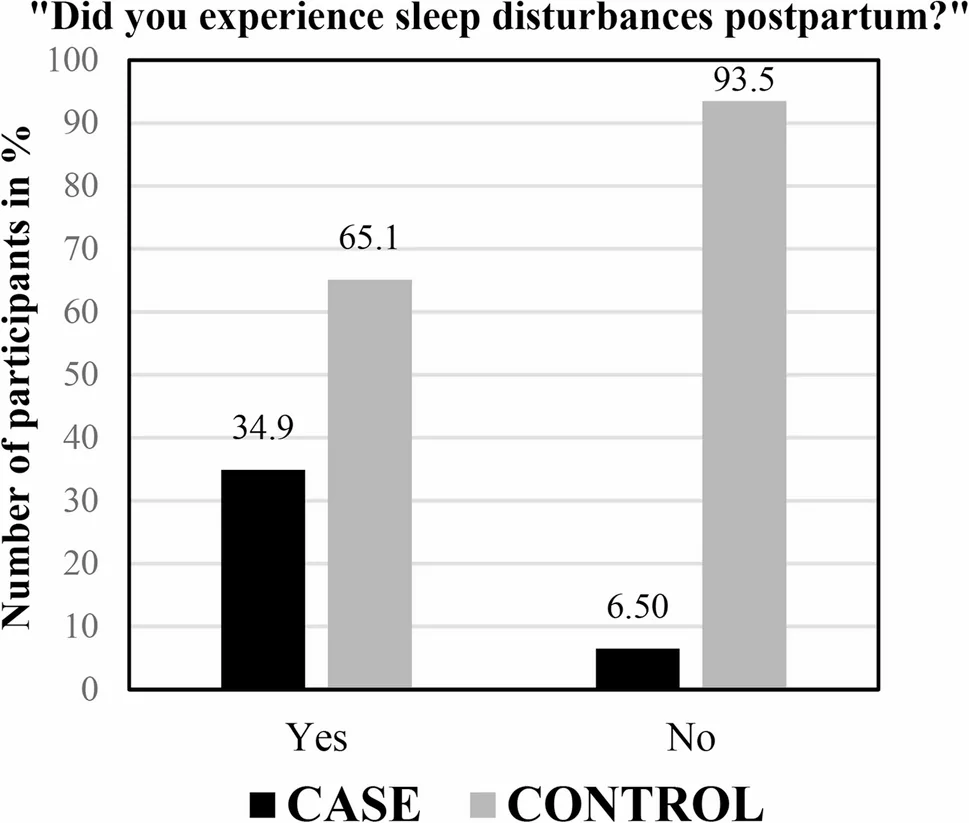
Sleep disturbances postpartum. Proportion of participants in the Case and Control groups reporting sleep disturbances during the first six weeks postpartum that were unrelated to the newborn’s sleeping pattern. Responses are presented as percentages within each group

In the first six weeks postpartum, 34.9% of participants in the Case group reported sleep disturbances unrelated to their newborn’s sleeping pattern, compared to 6.5% in the Control group (Fig. 9). A chi-square test indicated a significant difference between the groups (*p* < 0.05) (Table 8). The effect size of V = 0.34 showed a moderate significant association between group status and postpartum sleep disturbances.

**Table 8:** Associations between sleep disturbances postpartum and PPD

Variable	χ²	*p*-value	df	Cramer’s V
Chi-square test: sleep disturbances postpartum	119.68	7.44E-28	1	0.35

Association between sleep disturbances postpartum and PPD in the Case and Control groups were assessed using chi-square tests

*χ*² chi-square statistic, *df* degrees of freedom, effect sizes are reported as Cramer’s V

### Support postpartum

**Fig. 10:**
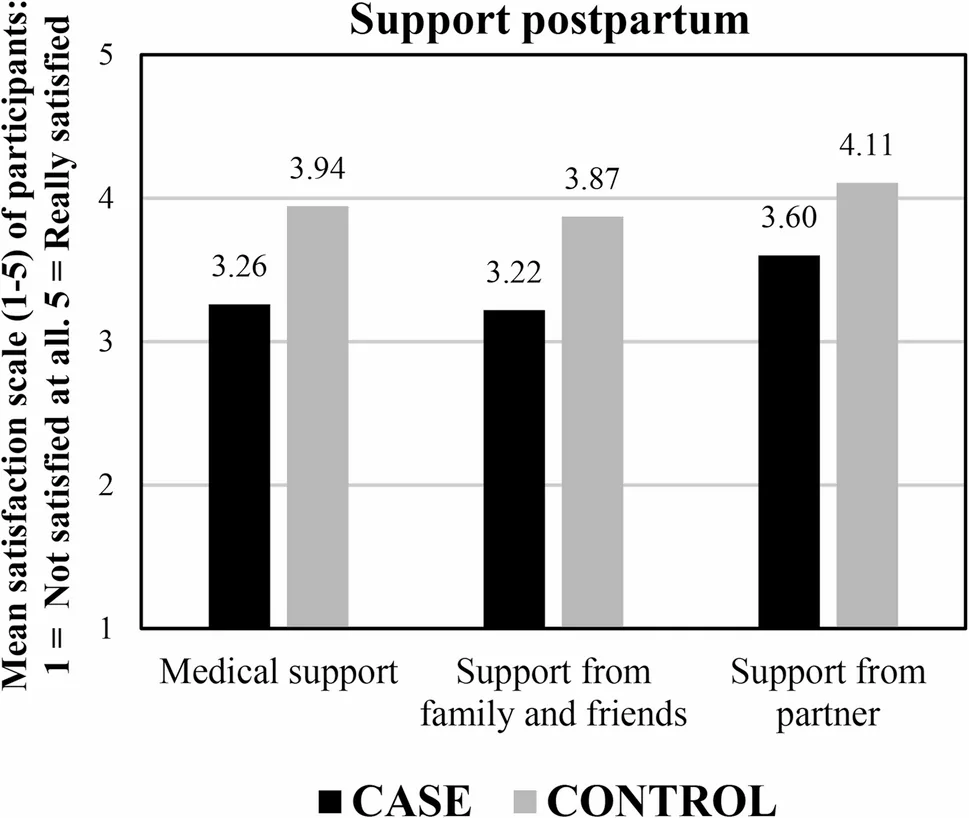
Mean satisfaction scores regarding postpartum support. Mean satisfaction scores for statements assessing satisfaction with medical, personal, and partner support postpartum among the Case and Control groups on a scale from 1 to 5 (1 = not satisfied at all, 5 = very satisfied). Higher scores indicate more satisfaction with each statement

A Likert scale question was used to examine participants’ satisfaction with received support during the first six weeks after birth, including medical, personal, and partner support (Fig. 10). Across all three statements, participants in the Control group reported higher satisfaction scores compared to the Case group (medical: 3.94 vs. 3.26; personal: 3.87 vs. 3.22; partner: 4.11 vs. 3.60). Chi-square analyses indicated significant group differences between Case and Control groups for all three support fields (medical personal and partner support) as shown by a *p-*value < 0.05. Effect size analyses (Cramer’s V) revealed a strong association between the group and satisfaction with medical and personal support, and a moderate association for support by one’s partner (Table 9).

**Table 9:** Association between satisfaction with support postpartum and PPD

Variable	χ²	*p*-value	df	Cramer’s V
Chi-square test: medical support postpartum	86.98	5.77E-18	4	0.30
Chi-square test: support from family and friends postpartum	86.33	7.94E-18	4	0.30
Chi-square test: support from partner postpartum	54.02	5.21E-11	4	0.24

Associations between support satisfaction postpartum and PPD in the Case and Control groups were assessed using chi-square tests. Analyses include: “Medical support postpartum”, “support from family and friends postpartum”, and “support from partner postpartum”

*χ*² chi-square statistic, *df* degrees of freedom, effect sizes are reported as Cramer’s V

#### Chronic stress postpartum

**Fig. 11:**
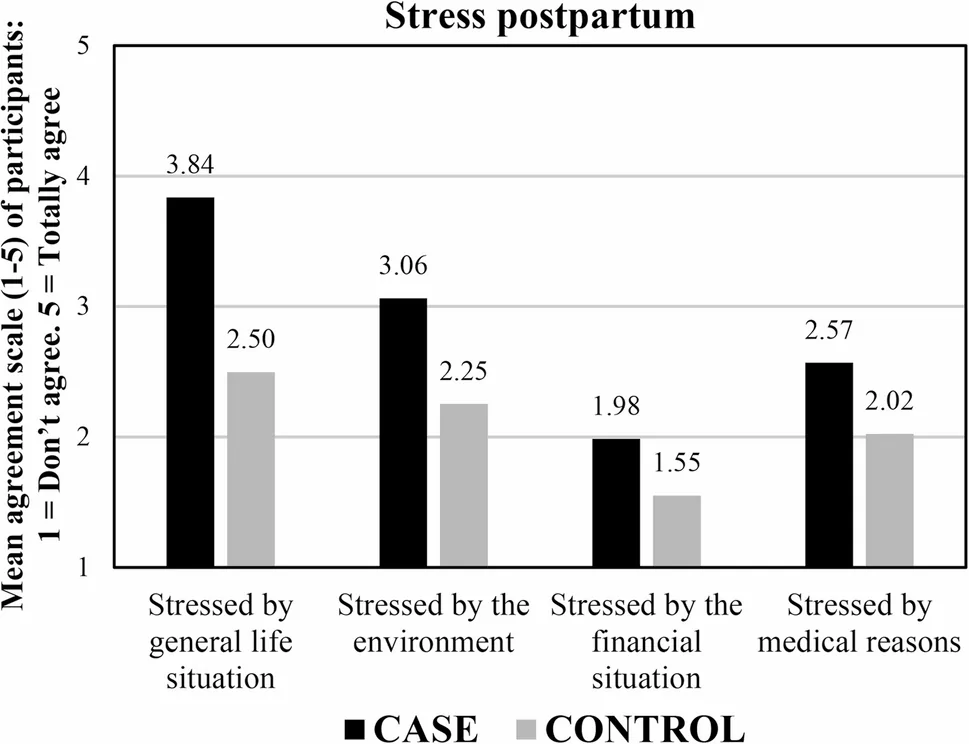
Mean agreement scores regarding chronic stress postpartum. Mean agreement scores for statements about chronic stress postpartum among the Case and Control groups are shown on a scale from 1 to 5 (1 = don’t agree, 5 = totally agree). Assessed statements include “stressed by general life situation,” “stressed by the environment,” “stressed by the financial situation,” and “stressed by medical reasons.” Higher scores indicate stronger agreement with each statement

The survey assessed chronic as well as sudden stress during the first six weeks postpartum. Chronic Stress during the postpartum period was assessed using Likert scale items ranging from 1 (don’t agree) to 5 (totally agree). As shown in Fig. 11, participants in the Case group reported higher average agreement scores across all statements regarding stress (I experienced stress because of - my general life situation; my environment, my financial situation, and medical reasons) compared to the Control group (3.84, 3.06, 1.98, and 2.57 vs. 2.50, 2.25, 1.55, and 2.02, respectively).

Chi-square analyses indicated significant group differences for all stress domains (*p* < 0.05). Effect size analysis (Cramer’s V) revealed strong associations for general life and environmental stress, and moderate associations for financial and medical stress (Table 10).

**Table 10:** Association between perceived stress postpartum and PPD

Variable	χ²	*p*-value	df	Cramer’s V
Chi-square test: stressed by general life situation	219.42	2.50E-46	4	0.48
Chi-square test: stressed by the environment	88.18	3.20E-18	4	0.30
Chi-square test: stressed by the financial situation	34.70	5.35E-07	4	0.19
Chi-square test: stressed by medical reasons	34.38	6.22E-07	4	0.19

Associations between stress postpartum and PPD in the Case and Control groups were assessed using chi-square tests. Analysed statements include: “Stressed by general life situation”, “stressed by the environment”, “stressed by the financial situation”, and “stressed by medical reasons”

*χ*² chi-square statistic, *df* degrees of freedom, effect sizes are reported as Cramer’s V

### Risk factors with no or weak significance

Several factors showed no significant associations with PPD during statistical analyses. These included pre-existing conditions such as polycystic ovary syndrome (PCOS), migraine, as well as pregnancy-related complications including thyroid dysfunction, gestational diabetes, preeclampsia, hyperemesis, and feeling more sick than usual during pregnancy. Obstetric factors such as birth mode, labour induction, birth arrest, premature birth, and postpartum conditions including hypertension, migraine, feeling more sick than usual postpartum, and inability to breastfeed were likewise not significantly associated with PPD. Episodic stress during pregnancy and the postpartum period also showed no significant associations.

Factors showing weak associations with PPD included family history of PPD, premenstrual syndrome (PMS), sleep disturbances during pregnancy, epidural anaesthesia, and several breastfeeding-related factors, including breastfeeding until the introduction of solid foods, early cessation of breastfeeding, dissatisfaction with the breastfeeding experience, and feelings of guilt regarding breastfeeding.

### Sensitivity analyses

Sensitivity analyses were conducted to examine whether associations differed depending on case definition. Women with self-reported PPD and women with EPDS scores ≥ 13 were analysed separately against the control group using the same Bonferroni-corrected significance threshold as applied in the primary analysis. Overall, most variables that showed moderate or strong associations in the primary analysis also remained statistically significant in both sensitivity analyses. Effect sizes were generally larger in the self-reported PPD subgroup than in the EPDS ≥ 13 subgroup. History of depression, prenatal depression, general stress during pregnancy, loneliness during pregnancy, postpartum sleep disturbances, support postpartum, and chronic stress postpartum showed the same classification of effect size in both sensitivity analyses as in the primary analysis. Several variables that demonstrated moderate associations in the primary analysis remained statistically significant in the self-reported PPD subgroup but did not reach the adjusted significance threshold in the EPDS ≥ 13 subgroup. These included perceived support during pregnancy, financial stress during pregnancy, satisfaction with birth, satisfaction with personal support during birth, satisfaction with medical support during birth, and stress related to medical reasons postpartum. Factors that were non-significant or showed only weak associations in the primary analysis remained non-significant or weak in the sensitivity analyses. No factor classified as non-significant in the primary analysis showed a moderate or strong association in either sensitivity analysis. Detailed results are presented in Table 11.

**Table 11:** Sensitivity analyses using alternative case definitions

Variable	Item	PPD vs. Control	EPDS ≥ 13 vs. Control
History of depression		*p* = 5.59E-18 V = 0.32**	*p* = 7.13E-08 V = 0.22**
Prenatal depression		*p =* 1.91E-32 V = 0.44***	*p* = 2.66E-13 V = 0.30***
Support during pregnancy	“I believed that support was available if needed”	*p* = 2.31E-07 V = 0.22**	*p* = 9.53E-02 V = 0.11
	“I was satisfied with the support I received”	*p* = 9.42E-06 V = 0.19**	*p* = 2.62E-04 V = 0.18**
Chronic stress during pregnancy	“I felt generally stressed”	*p* = 4.10E-10 V = 0.25***	*p* = 4.15E-11 V = 0.28***
	“I experienced financial stress”	*p* = 1.14E-04 V = 0.17**	*p* = 1.14E-03 V = 0.16
	“I was stressed by my environment”	*p* = 1.04E-05 V = 0.19**	*p* = 4.41E-04 V = 0.17**
	“I felt lonely”	*p* = 4.76E-16 V = 0.32***	*p* = 3.27E-12 V = 0.29***
Happiness during pregnancy		*p* = 2.44E-12 V = 0.28***	*p* = 1.33-06 V = 0.22**
Birth satisfaction	“I am satisfied about how the birth went”	*p* = 8.60E-14 V = 0.29***	*p* = 4.48E-02 V = 0.12
	“I was in control and able to make decisions autonomously”	*p* = 3.48E-14 V = 0.30***	*p* = 4.98E-04 V = 0.17**
	“I was satisfied with my personal support”	*p* = 2.25E-06 V = 0.20**	*p* = 3.73E-02 V = 0.12
	“I was satisfied with the medical support”	*p* = 2.10E-12 V = 0.28***	*p* = 1.97E-02 V = 0.13
Traumatic birth		*p* = 4.48E-18 V = 0.32**	*p* = 4.26E-05 V = 0.17*
Sleep disturbances postpartum		*p* = 1.25E-27 V = 0.39**	*p* = 1.96E-16 V = 0.31**
Support postpartum	Medical support postpartum	*p* = 1.41E-18 V = 0.34***	*p* = 2.65E-07 V = 0.23**
	Support from family and friends postpartum	*p* = 4.75E-17 V = 0.33***	*p* = 1.67E-07 V = 0.24**
	Support from partner postpartum	*p* = 4.89E-09 V = 0.24**	*p* = 8.14E-07 V = 0.22**
Chronic stress postpartum	“Stressed by general life situation”	*p* = 4.25E-43 V = 0.52***	*p* = 6.79E-18 V = 0.36***
	“Stressed by the environment”	*p* = 3.40E-16 V = 0.32***	*p* = 1.86E-09 V = 0.26***
	“Stressed by the financial situation”	*p* = 2.71E-07 V = 0.22**	*p* = 1.05E-03 V = 0.16**
	“Stressed by medical reasons”	*p* = 1.91E-08 V = 0.23**	*p* = 9.73E-03 V = 0.14

Women with self-reported PPD and women with EPDS scores ≥ 13 were analysed separately against the control group using chi-square tests. Reported values include p-value and effect size (Cramer’s V). Effect size classifications were based on thresholds in regard to degrees of freedom [29]. Variables without asterisks did not reach the Bonferroni-adjusted significance threshold (*p* < 0.000943)

* = weak association

** = moderate association

*** = strong association

## Discussion

### Key findings

The study investigated risk factors for PPD using a large sample of women from German-speaking countries. It identified multiple psychosocial and biological factors associated with PPD in a case-control comparison [1, 2, 7, 12–14, 24]. Women in the Case group consistently reported significantly higher stress levels during pregnancy and postpartum, including stress related to general life circumstances, the environment, medical issues, and financial concerns, showing a significant association between stress and PPD across the perinatal period [2, 12, 13, 35]. Satisfaction with support emerged as another critical factor, with women in the Case group being less satisfied with support from partner, family, and friends, as well as with medical care postpartum [2, 3, 12, 24]. Feelings of loneliness during pregnancy were higher in cases, reinforcing the role of social isolation as a psychosocial stressor [12, 13].

A history of depression and prenatal depressive symptoms also appear to be substantial risk factors for PPD [2, 3, 7, 24]. In this study, 24.2% of participants reported prenatal depression, suggesting that depressive symptoms may already be present during pregnancy in a substantial proportion of women. However, prospective studies are needed to determine whether these symptoms represent an early manifestation of PPD or a distinct antenatal depressive episode. Lower levels of happiness during pregnancy were also more common among cases, which may indicate that depressive symptoms may already be present during the antenatal period [1, 12, 24, 36].

Birth-related experiences also seem to associate with PPD. Women who reported experiencing a traumatic birth were more likely to belong to the Case group, and overall birth satisfaction was lower among cases, emphasising the association between subjective birth experience and PPD [35, 37, 38]. Medical aspects of childbirth, such as birth complications or birth mode (e.g., caesarean section) showed no significant association with PPD. This suggests that women’s subjective birth experience may be more closely associated with PPD in this dataset than objective medical factors [14, 24, 38].

Suffering from sleep disturbances postpartum was also significantly more common among cases in this study [2, 3, 24]. However, the directionality of this association remains uncertain, since the cross-sectional study design does not allow conclusions regarding whether sleep disturbances act as a risk factor for PPD or represent an early symptom of the disorder.

In summary, all shown factors, including chronic stress, dissatisfaction with perceived and received support, loneliness during pregnancy, history of depression, prenatal depression, reduced happiness during pregnancy, traumatic or negative birth experience, and postpartum sleep disturbances, present moderate to strong associations with PPD. These findings underscore the multifactorial nature of PPD and the broad range of factors associated with the disorder, with psychosocial factors appearing to show the strongest associations with PPD in this study [1, 12–14, 24, 39].

Several factors, particularly biological and obstetric factors, demonstrated only weak or non-significant associations with PPD. While these factors showed considerably smaller effect sizes than the psychosocial variables identified as the strongest correlates of PPD in the present study, they should not necessarily be dismissed as irrelevant. The absence of strong associations in the present study does not imply that these factors play no role in the development of PPD. Rather, the study design did not allow for the assessment of biological mechanisms such as hormonal changes, inflammatory markers, or HPA-axis functioning. Thus, while psychosocial factors appeared to be more influential in this dataset, biological predisposition may also be relevant but could not be directly assessed in this study [2, 3, 12]. Future studies, particularly those using prospective designs, are needed to further clarify the contribution of these factors to the development of PPD.

Sensitivity analyses were conducted to evaluate the case classification used in the present study. Women with EPDS scores ≥ 13 generally showed weaker associations than women with PPD. However, the overall pattern of associations remained largely comparable. For many of the factors identified in the primary analysis, the EPDS ≥ 13 group showed values that were intermediate between controls and women with PPD. This suggests that the EPDS-defined subgroup may represent women experiencing clinically relevant depressive symptoms that are less severe or less likely to have been formally recognised than those reported by women with a previous PPD diagnosis.

These findings are particularly relevant because many women with PPD remain undiagnosed. If case classification had relied exclusively on PPD diagnoses, women with elevated EPDS scores would have been included in the control group despite showing differences from controls across many of the factors examined in this study. Such an approach could have increased the risk of misclassification by grouping women with probable PPD symptoms together with women who did not report such symptoms. The sensitivity analyses suggest that women with EPDS scores ≥ 13 cannot be considered equivalent to controls, even though their associations were generally less pronounced than those observed among women with PPD. Consequently, including both groups within the primary case definition may have allowed the study to capture a broader spectrum of PPD symptomatology, including women whose symptoms may not have been formally recognised or diagnosed. This may be particularly relevant in population-based research, where a substantial proportion of women experiencing PPD remain undiagnosed and therefore may not be represented in studies relying solely on clinical diagnoses.

### Demographics and sample characteristics

A key strength of this study is the large and geographically concentrated sample. Nearly all participants resided in Germany, making this one of the few studies to provide data about PPD specifically for this population [15, 16]. The study sample was generally more highly educated and employed than the general population [40], indicating a slight selection bias, which is commonly observed in online survey research [18, 20]. Nevertheless, the findings still provide valuable insights into PPD within this demographic context of maternal health research [18–21].

### Strengths and limitations of the study

Using an online survey for the study allowed an effective and cost-efficient recruitment of a large sample size of 975 participants within a short period, consistent with established advantages of internet-based research methods [18–23]. The online survey achieved a high response rate (~ 60%) and completion rate (~ 85%), indicating strong engagement and reliability of the collected data, when compared with previous online studies [21–23]. By using an online survey, the study was able to cover a broad range of psychosocial and biological factors, providing a comprehensive evaluation of factors associated with PPD. The anonymity and convenience of online participation may have facilitated honest and thoughtful responses, thereby supporting data quality. This is also a key advantage frequently highlighted in the context of mental health research and online data collection [18, 21, 22]. Furthermore, the use of both self-reported PPD and EPDS scores enabled the identification of undiagnosed cases, strengthening the validity of the case-control classification and addressing the well-documented issue of underdiagnosis in PPD research, as well as in routine perinatal care [1, 5, 10, 11]. In addition, sensitivity analyses separating the case group into self-reported PPD cases and women with EPDS scores ≥ 13 showed overall similar patterns of association, indicating that the main findings remained generally consistent when the two case groups were examined separately.

Despite these strengths, the study has several limitations. The reliance on self-reported retrospective data introduces the possibility of recall bias, a common limitation in observational and survey-based studies [17, 20]. This is particularly relevant in participants with PPD, who may recall pregnancy and postpartum experiences more negatively compared to controls. Consequently, some associations may have been overestimated and should therefore be interpreted with caution. The use of a convenience sample recruited via online platforms introduces selection bias, resulting in a sample that is not representative of the general population. The sample was predominantly highly educated, employed, and residing in Germany, which limits the generalisability of the findings to women with lower socioeconomic status or limited digital access, who may also be at increased risk for PPD. In addition, recruitment via parenting apps and social media groups may have preferentially attracted women with a particular interest in pregnancy and postpartum health topics, potentially resulting in a more highly motivated sample. Consequently, the observed associations may not be fully generalisable to the broader population of postpartum women. In addition, the study design does not allow for adjustment of potential confounding variables, since all analyses were performed on a univariate level without multivariable modelling. Therefore, residual confounding between variables cannot be excluded, and the reported associations should be interpreted as potential risk markers rather than independent predictors. Further, the use of chi-square tests for Likert-scale variables does not account for the ordinal nature of these measures and may influence the sensitivity of effect estimates across multi-category response distributions. Another important limitation is that the questionnaire primarily assessed perceived psychosocial factors rather than objective measures of burden. Consequently, relevant personality traits, particularly neuroticism, were not directly assessed. Individuals with higher levels of neuroticism may perceive identical environmental, medical, or financial circumstances as more stressful than others and may also be more vulnerable to postpartum mental health symptoms [41]. Therefore, personality traits may represent an important unmeasured confounder. Furthermore, although the survey included a broad range of psychosocial and biological factors, biological mechanisms (e.g., hormonal changes) could not be directly measured, limiting the understanding of hormonal or other biological contributions to PPD [2, 3, 12]. Finally, the EPDS used for case classification was retrospectively adapted to fit the study design. Specifically, the recall period and item wording were modified to assess depressive symptoms occurring after childbirth over a period of up to five years, rather than symptoms experienced during the past seven days as in the original validated version. This adaptation could limit comparability with the original version.

## Conclusion

Overall, the findings highlight strong associations between psychosocial factors and PPD, particularly stress and lack of support during pregnancy, as well as perceived negative birth experiences, which is in line with existing systematic reviews and population-based studies [1, 7, 12–14, 24]. The study also suggests that depressive symptoms may already be present during pregnancy in a substantial proportion of women rather than emerging exclusively postpartum. This highlights the need for earlier detection and prevention strategies within routine antenatal care, especially since untreated PPD can lead to serious consequences for both mother and child [1, 2]. Implementing standardised screening tools such as the EPDS in routine gynaecological and perinatal healthcare settings could help identify at-risk women during pregnancy, enabling timely intervention before symptoms fully manifest in the postpartum period. Women identified as being at increased risk may particularly benefit from psychosocial support interventions and individualised support strategies [10, 11]. The study also demonstrates the utility of large-scale online surveys in efficiently collecting high-quality data across a broad population [18, 21, 22].

Future research should aim to integrate biological analyses with psychosocial evaluations to clarify the complex interplay associated with the onset of PPD, which can lead to more comprehensive prevention and treatment strategies in maternal and perinatal health care [2, 12]. In addition, future studies could not only investigate risk-associated variables but also focus on protective factors, including social support and emotional well-being, to further improve prevention strategies for PPD.

## Supplementary Information

Supplementary Material 1.

## Data Availability

The dataset and analysis code supporting the conclusions of this article are available in the OSF repository ([https://osf.io/akqpm]) under embargo and will be made publicly accessible upon publication. During the embargo period, the data are available from the corresponding author upon reasonable request.

## Abbreviations

CES-D: Center for Epidemiologic Studies Depression Scale
Cherries: Checklist for Reporting Results of Internet E-Surveys
EPDS: Edinburgh Postnatal Depression Scale
IBS: Irritable Bowel Syndrome
ICMJE: International Committee of Medical Journal Editors
PCOS: Polycystic Ovary Syndrome
PDSS: Postpartum Depression Screening Scale
PMS: Premenstrual Syndrome
PPD: Postpartum Depression
