# Supplementary material for: Identifying risk factors associated with postpartum depression: a retrospective case-control study
Source: BMC Pregnancy Childbirth. 2026 Sep 17;26:1027. doi: 10.1186/s12884-026-09932-2 (PMC13584356; doi:10.1186/s12884-026-09932-2)
Supplement: Supplementary file 1 [file 12884_2026_9932_MOESM1_ESM.docx]

**Additional files**

**Additional files legends**

**Additional file 1.** Literature-based risk factors for PPD assessed in the survey.
**Format:** Word document (.docx).
**Description:** Supplementary table summarising literature-based psychosocial and biological risk factors included in the online survey.

**Additional file 2.** Full survey (English version).
**Format:** Word document (.docx).
**Description:** Complete questionnaire containing all survey items and response options as presented to participants via SoSciSurvey.

**Additional file 3.** Interpretation of Cramer’s V effect size.
**Format:** Word document (.docx).
**Description:** Supplementary table outlining the interpretation of Cramer’s V effect sizes based on degrees of freedom, adapted from Cohen (1988)

**Additional file 4.** Sociodemographic distribution of the study sample.
**Format:** Word document (.docx).
**Description:** Supplementary figure showing the distribution of all participants by residence (A), occupational status (B), maternal age (C), and education (D).

**Additional file 5.** **Classification of analysed risk factors for PPD across the primary case-control analysis and sensitivity analyses.** Risk factors were classified as non-significant, weak, moderate, or strong according to Bonferroni-corrected chi-square tests and Cramer's V thresholds in regard to degrees of freedom. The primary analysis compared the combined Case group (self-reported PPD and EPDS ≥13) with the Control group, while sensitivity analyses compared women with self-reported PPD and women with EPDS scores ≥13 separately against the Control group. Associations classified as non-significant did not meet the Bonferroni-corrected significance threshold (p < 0.00094).

| Risk factor | References | Notes |
| --- | --- | --- |
| Polycystic ovary syndrome (PCOS) | Koric et al., 2021; Schoretsanitis et al., 2022 | - |
| Premenstrual syndrome (PMS) | Cao et al., 2020; Gastaldon et al., 2022 | - |
| Hormones | Kroll-Desrosiers et al., 2017; Payne and Maguire, 2019; Schiller et al., 2015; Stewart and Vigod, 2019; Yim et al., 2015; Yu et al., 2021 | - |
| Irritable bowel syndrome (IBS) | - | Hypothesised based on gut-brain axis and hormonal influences |
| Migraine | - | Hypothesised based on hormonal mechanisms (e.g., estrogen fluctuations) |
| Thyroid dysfunction | - | Hypothesised based on thyroid hormone dysregulation and hormonal impact |
| Hyperemesis | - | Hypothesised based on hormonal changes (hCG, estrogen, progesterone) |
| Gestational diabetes | Gastaldon et al., 2022; Huang et al., 2015; Silverman et al., 2017) | - |
| Birth mode + caesarean section | Beck-Hiestermann et al., 2024; Gastaldon et al., 2022; Xu et al., 2017; Zhao and Zhang, 2020 | - |
| Peridural anaesthesia (PDA) | Eckerdal et al., 2020; Lim et al., 2018 | - |
| Sleep disturbance | Koutra et al., 2018; Okun, 2016; Zhao and Zhang, 2020 | - |
| History of depression | Gopalan et al., 2022; Payne and Maguire, 2019; Silverman et al., 2017; Stewart and Vigod, 2019; Yu et al., 2021; Zhao and Zhang, 2020 | - |
| Family history of PPD | Klein et al., 2024 | - |
| Prenatal depression | Hofmeier et al., 2023 | - |
| Relationship satisfaction | Payne and Maguire, 2019; Yu et al., 2021 | - |
| Support | Payne and Maguire, 2019; Stewart and Vigod, 2019; Yim et al., 2015; Yu et al., 2021; Zhao and Zhang, 2020 | - |
| Stress | Chai et al., 2022; Jaramillo et al., 2023; Khoury et al., 2023; Payne and Maguire, 2019; Yu et al., 2021 | - |
| Birth complications | Jaramillo et al., 2023; Urbanová et al., 2021; Zhao and Zhang, 2020 | - |
| Birth satisfaction | Jaramillo et al., 2023; Urbanová et al., 2021; Zhao and Zhang, 2020 | - |
| Traumatic birth | Jaramillo et al., 2023; Beck-Hiestermann et al., 2024; Kountanis et al., 2020 | - |
| Breastfeeding | Henshaw, 2023; Koutra et al., 2018 | - |

**Additional file 1.**

**Additional file 2.**

**Additional file 3.**

| df | Neglectable association | Weak association | Moderate association | Strong association |
| --- | --- | --- | --- | --- |
| 1 | 0-0.1 | 0.1-0.3 | 0.3-0.5 | >0.5 |
| 2 | 0-0.07 | 0.07-0.21 | 0.21-0.35 | >0.35 |
| 3 | 0-0.06 | 0.06-0.17 | 0.17-0.29 | >0.29 |
| 4 | 0-0.05 | 0.05-0.15 | 0.15-0.25 | >0.25 |
| ≥5 | 0-0.04 | 0.04-0.13 | 0.13-0.22 | >0.22 |

**Additional** **file 4.**

**A**

**B**

**C**

**D**

Distribution of Participants by Place of Residence

Number of participants in %

|  | Country |
| --- | --- |
| Germany | 96.358 |
| Austria | 1.66493 |
| Switzerland | 1.66493 |
| Other country in the EU | 0.312175 |
| Country outside the EU | 0 |

Distribution of Occupational Status Among All Participants

Number of participants in %

|  | Occupation |
| --- | --- |
| Unemployed | 3.89744 |
| Working | 85.9487 |
| Care work | 3.28205 |
| In training (trainee, student) | 5.74359 |
| Pupil | 0.205128 |

Age Distribution of All Participants

Number of participants in %

|  | Age of the participant |
| --- | --- |
| 20 or younger | 0.923077 |
| 21-24 | 6.76923 |
| 25-29 | 24.2051 |
| 30-34 | 41.641 |
| 35-39 | 21.7436 |
| 40 or older | 4.61538 |

Distribution of Education Levels Among All Participants

Number of participants in %

|  | Education level |
| --- | --- |
| No degree | 0 |
| Secondary school diploma | 7.48718 |
| Abitur | 8.20513 |
| Completed vocational training | 30.7692 |
| University degree | 53.2308 |

**Additional file 5.**

| Variable | Item | Case vs Control | PPD vs Control | EPDS ≥13 vs Control |
| --- | --- | --- | --- | --- |
| History of depression |  | Moderate | Moderate | Moderate |
| Family history of depression |  | Weak | Weak | Weak |
| Conditions before pregnancy | PCOS | Non-significant | Non-significant | Non-significant |
|  | IBS | Non-significant | Weak | Non-significant |
|  | PMS | Weak | Weak | Non-significant |
|  | Migraine | Non-significant | Non-significant | Non-significant |
| Conditions during pregnancy | Prenatal diabetes | Non-significant | Non-significant | Non-significant |
|  | Migraine | Non-significant | Non-significant | Non-significant |
|  | Hyperemesis | Non-significant | Non-significant | Non-significant |
|  | Preeclampsia | Non-significant | Non-significant | Non-significant |
|  | Thyroid dysfunction | Non-significant | Non-significant | Non-significant |
|  | Sleep disturbances | Weak | Weak | Weak |
|  | More sick than usual | Non-significant | Non-significant | Non-significant |
| Prenatal depression |  | Strong | Strong | Strong |
| Support during pregnancy | “I believed that support was available if needed” | Moderate | Moderate | Non-significant |
|  | “I was satisfied with the support I received” | Moderate | Moderate | Moderate |
| Chronic stress during pregnancy | “I felt generally stressed” | Strong | Strong | Strong |
|  | “I experienced financial stress” | Moderate | Moderate | Non-significant |
|  | “I was stressed by my environment” | Moderate | Moderate | Moderate |
|  | “I felt lonely” | Strong | Strong | Strong |
| Episodic stress during pregnancy |  | Non-significant | Non-significant | Non-significant |
| Happiness during pregnancy |  | Strong | Strong | Moderate |
| Birth mode |  | Non-significant | Non-significant | Non-significant |
| Birth satisfaction | “I am satisfied about how the birth went” | Moderate | Strong | Non-significant |
|  | “I was in control and able to make decisions autonomously” | Moderate | Strong | Moderate |
|  | “I was satisfied with my personal support” | Moderate | Moderate | Non-significant |
|  | “I was satisfied with the medical support” | Moderate | Strong | Non-significant |
| Interventions during birth | Labour drip | Non-significant | Non-significant | Non-significant |
|  | Epidural anaesthesia | Weak | Non-significant | Non-significant |
|  | Birth arrest | Non-significant | Non-significant | Non-significant |
|  | Premature birth | Non-significant | Non-significant | Non-significant |
| Traumatic birth |  | Moderate | Moderate | Weak |
| Conditions postpartum | Hypertension | Non-significant | Non-significant | Non-significant |
|  | Migraine | Non-significant | Non-significant | Non-significant |
|  | Sleep disturbances postpartum | Moderate | Moderate | Moderate |
|  | More sick than usual | Non-significant | Non-significant | Non-significant |
| Breastfeeding until solid foods |  | Weak | Weak | Non-significant |
| Breastfeeding difficulties | “I couldn't produce enough milk” | Non-significant | Weak | Non-significant |
|  | “I couldn't breastfeed at all” | Non-significant | Non-significant | Non-significant |
|  | “I stopped earlier than I wanted” | Weak | Weak | Non-significant |
|  | “I am unsatisfied with my breastfeeding experience” | Weak | Weak | Weak |
|  | “I feel guilty about my breastfeeding experience” | Weak | Weak | Weak |
| Support postpartum | Medical support postpartum | Strong | Strong | Moderate |
|  | Support from family and friends postpartum | Strong | Strong | Moderate |
|  | Support from partner postpartum | Moderate | Moderate | Moderate |
| Chronic stress postpartum | “Stressed by general life situation” | Strong | Strong | Moderate |
|  | “Stressed by the environment” | Strong | Strong | Moderate |
|  | “Stressed by the financial situation” | Moderate | Moderate | Moderate |
|  | “Stressed by medical reasons” | Moderate | Moderate | Non-significant |
| Episodic stress postpartum |  | Non-significant | Weak | Non-significant |
